# The Clinical Spectrum of Resistance to Thyroid Hormone Alpha in Children and Adults

**DOI:** 10.4274/jcrpe.galenos.2020.2019.0190

**Published:** 2021-02-26

**Authors:** İbrahim Mert Erbaş, Korcan Demir

**Affiliations:** 1 Dokuz Eylül University Faculty of Medicine, Department of Pediatric Endocrinology, İzmir, Turkey

**Keywords:** Constipation, developmental delay, growth failure, central hypothyroidism, autism spectrum disorder, LT4, impaired sensitivity to thyroid hormone

## Abstract

Resistance to thyroid hormone alpha occurs due to pathogenic, heterozygous variants in *THRA*. The entity was first described in 2012 and to date only a small number of patients with varying severity have been reported. In this review, we summarize and interpret the heterogeneous clinical and laboratory features of all published cases, including ours. Many symptoms and findings are similar to those seen in primary hypothyroidism. However, thyroid-stimulating hormone levels are normal. Free triiodothyronine (T3) levels are in the upper half of normal range or frankly high and free thyroxine (T4) levels are low or in the lower half of normal range. Alterations in free T3 and free T4 may not be remarkable, particularly in adults, possibly contributing to underdiagnosis. In such patients, low reverse T3 levels, normo- or macrocytic anemia or, particularly in children, mildly elevated creatine kinase levels would warrant THRA sequencing. Treatment with L-thyroxine results in improvement of some clinical findings.

## Introduction

The thyroid gland has important roles in energy homeostasis, skeletal growth, cardiac and gastrointestinal function, and maturation of the central nervous system (1). Thyrotropin-releasing hormone (TRH) produced by the hypothalamus stimulates the pituitary gland to release thyroid-stimulating hormone (TSH), which results in synthesis and secretion of thyroid hormones (TH) from the thyroid. The term TH comprises T4 (thyroxine, a prohormone and the predominant product of thyroid) and T3 (tri-iodothyronine, the bioactive hormone). A negative-feedback mechanism provides balance between TH levels and TRH-TSH production (2).

TH enter cells via a number of membrane transporters, including tissue specific entities such as monocarboxylate transporter 8 (MCT8) in the central nervous system (3). Intracellular deiodinase enzymes regulate TH concentrations and convert T4 to T3 and various metabolites (4). T3 binds nuclear receptor proteins and regulates target gene transcription. In the absence of T3, receptor-protein complexes repress basal gene transcription (5). There are two types of TH receptor (TR): alpha (TRα) and beta (TRβ). These receptors are highly homologous and encoded by the genes *THRA* (chromosome 17) and *THRB* (chromosome 3), respectively. TRα has two isoforms produced with alternative splicing. TRα1 is mainly expressed in the central nervous system, bone, myocardium, skeletal muscle and gastrointestinal tract, while TRα2 is expressed in various tissues but has no binding site for T3 and thus its function is enigmatic (6,7). TRβ1 is predominantly expressed in liver, kidney, thyroid gland, brain, pituitary, and inner ear. TRβ2 expression is limited to the hypothalamus, pituitary gland, inner ear and retina, and plays the main role in the hypothalamic-pituitary-thyroid (HPT) axis (6,7,8).

Variants in TR genes cause particular forms of resistance to TH (RTH) (9). The first instance of this disease spectrum was reported by Refetoff et al (10) in 1967. However, demonstration of the underlying genetic defect in *THRB* took more than two decades (11). Pathogenic variants in *THRB* result in RTH beta (RTHβ, dominant OMIM #614450 and recessive OMIM #274300). The incidence of RTHβ is reported to be approximately 1/40000 and is characterized by goiter, tachycardia, hyperactivity, failure to thrive and cognitive impairments with high serum TH levels, but normal or mildly elevated TSH (12,13,14). The first case of TH resistance in TRα (RTHα, OMIM # 614450) due to a pathogenic, heterozygous variant in *THRA*, was published in 2012 by Bochukova et al (15). To date, 40 cases (13 adults, 27 children) from 28 different families with 25 different variants in *THRA* gene have been published (Tables 1, 2) (15,16,17,18,19,20,21,22,23,24,25,26,27,28,29,30,31,32).

The main symptoms and findings of RTHα include varying degrees of constipation, developmental delay, growth failure, and anemia, which are associated with the tissues where TRα is the main TR and are common to both primary hypothyroidism and RTHα. In the former, there is inadequate TH to induce TRα while reduced activity of TRα is the mechanism in the latter (33). Furthermore, there are interesting additional features in some of the cases with RTHα including skin tags (18,19,25), epilepsy (18,23), and the individual clinical picture or laboratory findings becoming less remarkable with age (17,24). The disease is thought to be underdiagnosed, given that serum TH levels are not distinctive as is seen in RTHβ, and TSH is not elevated since TRβ is intact (33,34).

## Genetics

To date, 25 different variants in *THRA* have been published (Tables 1, 2). Six variants were inherited from an affected parent. Three of the 25 variants were frameshifts, which affected four cases more severely (16,18,24). Three distinct variants resulted in a premature stop codon (21,28,31). However, most of the variants in *THRA* were missense mutations (15,19,20,21,22,23,24,25,26,29,30,32). All of the RTHα patients were heterozygous for the variant, showing that mutant TRα had a dominant-negative effect on the wild-type receptor, in a similar fashion to RTHβ (33). It should be noted that some of the variants have not been functionally characterized (20,21,26,27,28,29,31). In addition, one of the variants (c.1044G>T) found among subjects with autism spectrum disorder was a synonymous substitution (26).

The reported cases showed that there was a genotype-phenotype correlation in patients with RTHα. The most severe cases tended to have frameshift variants, but missense variants usually caused a milder phenotype (18,21,24). In addition, patients with the same variants in *THRA* can present with different clinical phenotypes, suggesting that additional factors, possibly cofactor proteins, affect TH activity (35).

It was reported that, in the presence of high T3 levels, mutant TRα can exhibit some degree of transcriptional activity, in a similar fashion to the wild-type receptor. This finding suggests that increased circulating T3 levels might have some benefit in ameliorating the dominant-negative activity of mutant TRα, although it is not clear whether high levels of T3 are a result of a compensatory mechanism (19,23,24). With the exception of one case with a mutation in both TRα1 and 2, who presented with severe atypical malformations (22), similar clinical features have been observed due to variants affecting either TRα1 alone or TRα1/2 (33).

## Pathophysiology

The mutant TRα behaves as a dominant-negative repressor of T3 target gene expression in RTHα and also inhibits the function of wild-type TR (15). TRα and TRβ act via transcriptional repressors, such as nuclear receptor corepressor-1 (NCoR1), in the absence of T3. This effect results in modification of histone deacetylase (HDAC) enzymes into a co-repressor complex, which suppresses basal T3 target gene transcription with remodeling of chromatin (36). When T3 binds to its receptors, a structural change is initiated, which results in disruption of TR and NCoR1. Furthermore, modification of nuclear receptor coactivators initiate the expression of T3 target genes (37,38).

If TRα is mutant, it cannot release NCoR1 as a response to T3. Consequently, T3 target gene transcription remains suppressed because of the inhibition of wild-type TR through constant HDAC-induced chromatin remodeling. In the light of this molecular information, RTHα demonstrates clinical features with reduced T3 action in related tissues. In addition, a dominant-negative potential of the mutant TRα determines the severity of disease (38).

## Clinical Features

The first experimental study of TRα was reported in 1997, 15 years before the first human cases were reported, showing that a TRα knock-out mouse had postnatal growth arrest with delayed maturation in small intestine and bones (39).

Data regarding physical features of patients with RTHα are generally limited and heterogeneous in the published reports. No descriptive data were given for seven children who were shown to have *THRA* variants during genetic analyses for autism spectrum disorder (20,26). The clinical features and underlying mechanisms, mainly derived from animal studies, are summarized in Table 3.

## Appearance

Patients with RTHα are usually born after an uneventful pregnancy (33). In severe cases, macroglossia, coarse facial features, and umbilical hernia have been noted in early infancy (18,24,32). However, there were also two children with no suggestive symptoms or clinical findings associated with hypothyroidism, who were diagnosed by family screening (24).

Coarse face including macroglossia, flattened large nose, thick lips, deep voice, and hoarse cry are the common features in nearly one third of the patients with RTHα (15,16,18,19,21,22,23,24,25,28,29,30,31,32). In addition, micrognathia and/or hypertelorism were reported in several cases (21,22).

Rough and dry or thickened skin, reflecting hypothyroidism, has been reported particularly in children in contrast to adult cases (16,21,28,31). In mice with mutant TRα, tissue iodothyronine deiodinase (DIO) 3 levels were reduced (40). In addition, topical inhibition of DIO3 enzyme was demonstrated to increase keratinocyte proliferation in animal models (40,41). Therefore, dermal symptoms in TRα patients are thought to be related to a similar mechanism. Skin tags were present in 21% of cases with RTHα; seven among 33 cases with available data (18,19,24,25). Bilateral inguinal hernia and umbilical hernia were reported in two children (25,29).

## Skeletal Findings

Skeletal manifestations such as growth retardation, patent cranial sutures, epiphyseal dysgenesis, and delayed dental eruption have been demonstrated in mice with mutant TRα1 receptor (42,43). In addition, mice with *THRA* variant presented with decreased endochondral and intramembranous ossification, with retarded closure of skull sutures (44). Delayed ossification in these animal models caused impaired bone remodeling and thus short stature with skeletal deformities. However, bone strength was normal, which may explain why pathologic fractures are not seen in humans with RTHα (43). Further molecular studies demonstrated that mutant TRα caused reduced transcription of target genes including growth hormone receptor, insulin-like growth factor-1 (IGF-1) or its receptor and fibroblast growth factor receptor-1 or -3. Moreover, decreased signaling in post-receptor pathways in osteoblasts or chondrocytes was reported (45,46,47,48,49,50).

Short stature is one of the most common clinical findings in children with RTHα (12 among 20 children with available data, 60%). Ten of the 12 short children did not receive L-thyroxine (LT4) therapy before diagnosis and the lowest height standard deviation (SD) score was -3.1 (15,16,21,23,24,25,28,29). A previously untreated, three years and 11 months old Chinese female was reported with a height of 85.5 cm but the SD score was not provided (31). All of the remaining eight children with normal height had missense variants. Six of them (85.7%) had a height SD score between -1.66 and 0 and none of them had received any treatment. Half of the 12 adult cases with available data had normal height, the tallest being 186 cm. All of them had missense variants and three had received LT4 starting from childhood (16,18,19,21,22,23,24).

Wormian bones in skull sutures were present in 10 among 31 cases with available data (32%) (15,24,25). Various other skeletal deformities, including delayed bone age, genu valgum, coxa valga, short tubular hand bones, late closure of fontanelles, and femoral epiphyseal dysgenesis were also reported (15,19,21,22,24,25,28,29,31). Mesomelic shortening of upper and lower limbs cause increased sitting/total height ratio (21,24,25). Skull radiography showed cranial hyperostosis in some patients (18,19,24). Espiard et al (22) reported a 27 years-old case with RTHα, who had severe deformities resembling cleidocranial dysplasia (clavicular agenesis, humero-radial synostosis, syndactyly of toes, agenesis of the 12^th^ ribs and scoliosis). However, these findings were atypical for RTHα and have not been reported in any other case to date. Bone mineral density was reported to be normal in three adult patients (19).

Normally, tooth eruption is expected to occur before 13 months of age (51). Delayed tooth eruption was detected in eight among 18 children with available data (44%) (15,24,25,29).

Bochukova et al (15) reported a mild hypermobility and ligamentous laxity in ankles and knees. Although muscle tone was decreased in some cases with RTHα, their muscle strength was almost normal (15).

## Neuromotor Development

T3 and its receptors play a major role in neuronal migration, synaptogenesis, maturation, myelination and differentiation of oligodendrocytes or glial cells (52). That is why TRα knockout animals showed a severe delay in postnatal development and locomotor dysfunction (53). TRα disruption had significant effects on cerebellar formation and hippocampal functions and TRα mutant mouse models had reduced brain mass (54,55,56). Wilcoxon et al (57) demonstrated behavioral inhibition and decreased learning and memory function in mice lacking all isoforms of TRα.

In infants with RTHα, delayed milestones for motor and speech abilities are the most common symptoms, noted in 34 among 40 cases (85%) (15,16,18-21,23,24,25,26,28,29,30,31,32). Reduced IQ, notable impairments in cognitive functions, slow motion movements, evident motor discoordination including dyspraxia, ataxia, and broad or unstable gait are some of the clinical findings on neurological examination (15,18,19,24,28,32). Remarkably, two cases with the A263V variant were able to attend university without LT4 treatment (Demir-unpublished observation of Patient 3.III.1 in reference 24,25). The first patient had no symptoms and was detected during family screening (24). The second case had mild delay in motor and mental development during childhood and received little teaching support (25). Axial hypotonia and slow motor development can also be seen (23). Clumsiness due to motor discoordination and difficulty with fine motor abilities has been reported in some patients, who were incapable of writing or drawing (15,18,28). Speech delay and dysarthric or slow speech are significant disabilities and are seen in the majority of cases (15,16,18,19,21,23,24,28). Macrocephalia is also a common clinical finding (23 among 33 cases with available data, 70%) (15,16,18,19,20,21,22,23,24,25,29,30,31).

Furthermore, Demir et al (24) reported a 35-year-old adult case, whose developmental delay during childhood was more remarkable compared to her affected son. As an adult, she presented with an attenuated clinical picture including mild intellectual deficit, no cardiac problems, and normal thyroid function tests, despite not being treated. Similar observations were also made in a mouse model with a heterozygous TRα1 variant at the same position (53,58). These mice showed severe but transient impairment of postnatal development and growth. The mechanisms underlying the amelioration of deficits caused by these TRα1 variants with age are unknown.

Seizures after stimulation with light or audio and abnormal evolution of GABAergic neurons in TRα1 mutant mice correlated with epilepsy in human cases (42,59,60). To date, three cases with RTHα were reported to be suffering from epileptic seizures in childhood (18,23,32).

A notable anxiety in unfamiliar environments and reduced cognitive functions were observed in TRα1 mutant animal models (59). Another study demonstrated that TRα1 mutant mice developed depressive and anxiety behaviors (61). Kalikiri et al (26) investigated 30 children diagnosed as autism spectrum disorder and found *THRA* variants in six of them. Unfortunately, no additional clinical data regarding these children were provided. Coexistence of autism spectrum disorder and RTHα was reported in two more patients, suggesting that RTHα should be excluded in patients with autism spectrum disorder (20,31).

## Constipation

TRα is the dominant TR in the intestinal tract (6,7). In a study with TRα1 mutant mice models, shortened villi, increased differentiation in crypt cells and decreased stem cell proliferation were observed (62). Independent of age, constipation is one of the most common clinical symptoms in human cases, being reported in 26 among 31 cases with available data (84%) (15,16,18,19,21,23,24,25,28,29,31,32). The atypical patient reported by Espiard et al (22), was the only patient to develop chronic diarrhea, at the age of 12. Abdominal radiographs showed dilated bowels. Decreased peristalsis was also observed by colonic manometry in several cases with RTHα (15,18).

## Cardiovascular System

TRα1 is expressed in myocardium and it was suggested to be responsible for cardiac myoblast differentiation in experimental studies (63). Mutant TRα1 mice models showed symptoms in the cardiovascular system associated with hypothyroidism, such as bradycardia or weak cardiac contractions (64). Makino et al (65) found that the predominant TR in mouse coronary smooth muscle cells was TRα, and suggested that coronary vascular tone was regulated by TRα. However, cardiac pathologies or symptoms do not seem to be common in humans with RTHα. Although most of the patients had normal heart rate or blood pressure, some cases were reported to have bradycardia (15,18,19). At the time of writing, three cases with cardiomyopathy and one case with pericardial effusion have been reported (21,24).

## Metabolic Problems and Fertility

TRα null or mutant mice had lower core body temperature due to impaired facultative thermogenesis (66). Although most of the animal models with mutant TRα were thin, several studies described obesity (58). In the same study, it was also reported that the TRα1 R384C mutant mice were hyperphagic but resistant to obesity (58). It was suggested that hypermetabolism, mediated centrally through apo-TRα1 resulted in reduced adipose tissue and lower body weight (67). However, eight among 33 humans diagnosed as RTHα with available data (24%) were obese and six of them were adults (15,18,23,24). Low resting energy expenditure (metabolic rate) was also reported in some patients with RTHα (15,18,19,22). In addition, total cholesterol and low-density lipoprotein (LDL) levels were high in several patients (16,18,19).

As RTHα can be seen in children of affected adults, it suggests that fertility might be unaffected in either gender. Regular pregnancies after spontaneous conception were reported, in even moderately affected and untreated female RTHα cases (24). Only one patient had late-onset of puberty and menarche at 16 years-old, with normal gonadotropin and estrogen levels (18).

## Laboratory

Unfortunately, relevant measurements were inconsistently reported in the published cases and so data is incomplete for all the case reports. In addition, while the majority of available data in the literature were presented as exact values with their reference ranges, some reports included only categorized data (Tables 1, 2).

## Thyroid Function Tests

Thyroid function tests of individuals suspected of having RTHα should be cautiously interpreted since the literature data were derived from cases with varying severity of RTHα and from different age groups. Abnormal TH levels are more likely to be found in severe cases and in children. Since the TH and TSH levels seem to differ if there has been previous LT4 use, we chose to evaluate the data from the cases who had not received LT4 previously (LT4-naive) separately from the patients who were analyzed after discontinuation of LT4 treatment.

## Individuals Who had not Receive Any Thyroid Hormone

A normal neonatal congenital hypothyroidism screening result [total T4 62 nmol/L (-1.3 SD), TSH 1 mIU/L] was reported in a case with RTHα, who also had an uneventful neonatal period (23).

**Figure 1:**
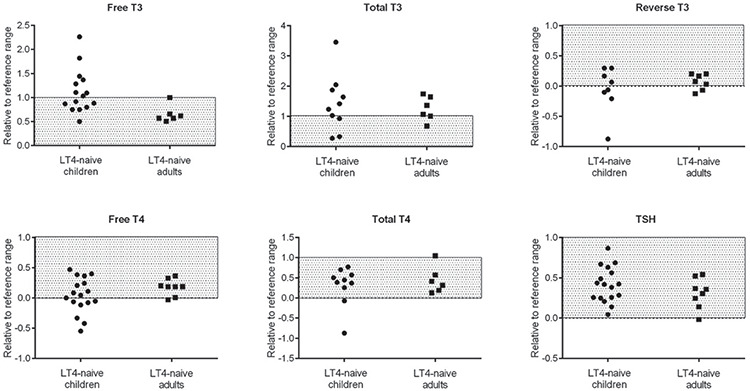
Thyroid function test results in previously untreated children and adults [derived from all available data in Table 1 (Cases 1-10, 14, 17-20, 24, and 25) and Table 2 (Cases 1-5, 9, 11, and 13)]. All of the data (x) was expressed relative to the relevant reference range with the following formula: (x - lower limit of normal range) / (upper limit of normal range - lower limit of normal range). Grey shaded areas indicated the normal range LT4: L-thyroxine

TSH levels were all normal in affected children. Among the adult patients, an atypical case with severe malformations was the only one with abnormal TSH (0.343 mIU/L, normal range 0.4-3.6) (Figure 1) (22).

Differences of TH levels among treatment-naive children and adults are also shown in Figure 1. All of the free T3 (fT3) and the majority of total T3 levels were in the upper half of normal range or frankly elevated. Elevated fT3 levels were found only in treatment-naive children but not in such adult cases. All of the free T4 (fT4) and the majority of total T4 levels were below the reference range or in the lower half of the normal range. Low fT4 concentrations were more frequently present among children. In adult patients, fT4 levels were all normal, except for one case (30).

**Figure 2:**
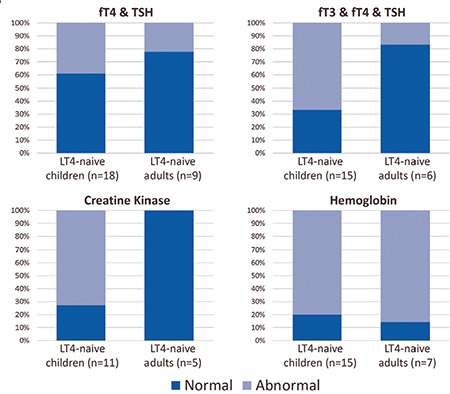
Classification of thyroid hormone profiles and peripheral indicators of hypothyroidism belonging to previously untreated children and adults [derived from all available data in Table 1 (Cases 1-10, 14, 17-21, 24, and 25) and Table 2 (Cases 1-5, 9, and 11-13)] LT4: L-thyroxine, fT3: free T3, fT4: free T4, TSH: thyroid-stimulating hormone

Both fT4 and TSH were normal in 61% (11 among 18) of children and 78% (7 among 9) of adults. Normal fT3, fT4 and TSH were noted in 33% (5 among 15) and 83% (5 among 6) of children and adults, respectively (Figure 2). In such cases, a high T3/T4 ratio or low or low-normal reverse T3 (rT3) level, resulting in an increased T3/rT3 ratio can be suggestive of RTHα (33). These abnormalities in RTHα patients may be the result of changes of DIO1 and DIO3 levels in tissues, as the expression of both are regulated by TRα. In a study, TRα1 mutant mice had raised hepatic DIO1 levels, which converts T4 to T3 (42). Therefore, this finding was related to high T3 levels and an increased T3/T4 ratio in RTHα. In addition, decreased DIO3 levels in tissues may result in low rT3 levels, causing reduced inner-ring deiodination of T4 to rT3 (40).

## Individuals Who Discontinued Treatment

After cessation of LT4 treatment, mildly elevated TSH may be seen, as was reported in one adult and one child with RTHα (17,18). The child, in whom TSH rose at the age of 11 after discontinuation of LT4, had normal pretreatment TSH levels at 5 and 6 years of age (17). In contrast, TSH remained in the normal range in three adult patients and an adolescent case (19,25) after LT4 cessation. Off thyroxine treatment, patients had marginally low or low-normal fT4. A wide range of free or total T3 data (varying from the lower half of the normal range to elevated levels) was reported. Nevertheless, rT3 levels were all low (17,18,19,25).

## Individuals Receiving Thyroid Hormone

Under LT4 treatment, fT3 and fT4 levels increased in patients with RTHα, while TSH was suppressed, a similar pattern to that found during the treatment of central hypothyroidism (15,17,18,19,23,24,29). One patient with atypical phenotype was treated with liothyronine, which caused a rise in fT3 level, suppressed TSH level, and markedly reduced fT4 concentration (22).

## Anemia

The relationship between anemia and hypothyroidism is well-known (68). Animal models lacking TRα demonstrated compromised erythropoiesis (69,70). In a study by van Gucht et al (71) of progenitor cells derived from RTHα patients, it was shown that these cells differentiated more slowly than controls. In humans, 23 among 30 cases with available data (77%) had anemia, and it has been one of the most common findings in humans with RTHα (16,18,19,21,22,23,24,25,28,29,31,32). The rate of anemia was similar between treatment-naive children (80%) and adults (86%) (Figure 2). In the reports where exact values were included, hemoglobin levels ranged between 8.6-10.9 g/dL and 9.6-12.9 g/dL in children and adults, respectively. In the majority, anemia was normocytic and normochromic; macrocytic anemia was described in three cases (13%) (15,18,22).

An increase in serum levels of interleukin-8 (IL-8), a pro-inflammatory cytokine, was shown in RTHα patients. However, neutrophil or macrophage functions, which are partly mediated by IL-8, were found to be normal in those cases (72).

## Other Biochemical Findings

Both thyroglobulin and urinary iodine levels are expected to be in the normal range (34). Similar to primary hypothyroidism, high total cholesterol and LDL levels, and low or low-normal levels of IGF-1 can be found in RTHα (33,34).

**Figure 3:**
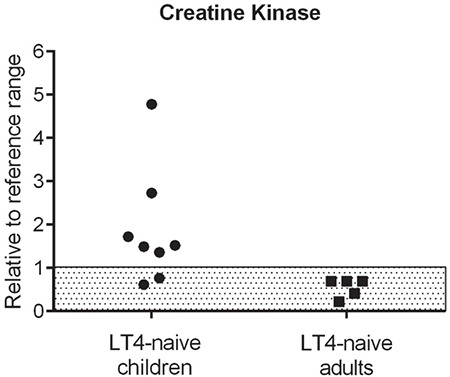
Numerical values of creatine kinase levels obtained from previously untreated children and adults with resistance to thyroid hormone alpha [derived from all available data in Table 1 (Cases 3-7, 10, 17, and 20) and Table 2 (Cases 3-5, 9, and 11)]. All of the data (x) was expressed relative to the relevant reference range with the following formula: (x - lower limit of normal range) / (upper limit of normal range - lower limit of normal range). Grey shaded area indicated the normal range LT4: L-thyroxine

In primary hypothyroidism, creatinine kinase (CK) can also be elevated (73). Human data demonstrate that CK might be a promising biomarker for diagnosis of RTHα, particularly in children. Eight among 11 treatment-naive children (73%) with available data had elevated CK levels (range; 218-981 U/L; 1.3-4.36 times upper limit of normal), while all of the treatment-naive adults with available data (n=5) had normal CK levels (Figures 2 and 3) (15,16,22,22,23,24,25,28,29,31). In contrast, elevated CK levels were noted in three of four adult patients (364-387 U/L; 1.90-2.02 times upper limit of normal) and in the two children (196-213 U/L; 1.03-1.31 times upper limit of normal) who were assessed after discontinuation of LT4 (17,18,19,25).

Recently, Boumaza et al (74) reported that biofluids (urine and plasma samples) of TRα-mutant mice showed distinct metabolomic profiles from controls, including increased urinary levels of hippurate and decreased urinary levels of isovalerylglycine, dimethylamine, trimethylamine, and choline. They suggested that easily accessible nuclear magnetic resonance-based metabolic fingerprints of biofluids could be used to diagnose RTHα in humans (74).

## Differential Diagnosis

**Figure 4:**
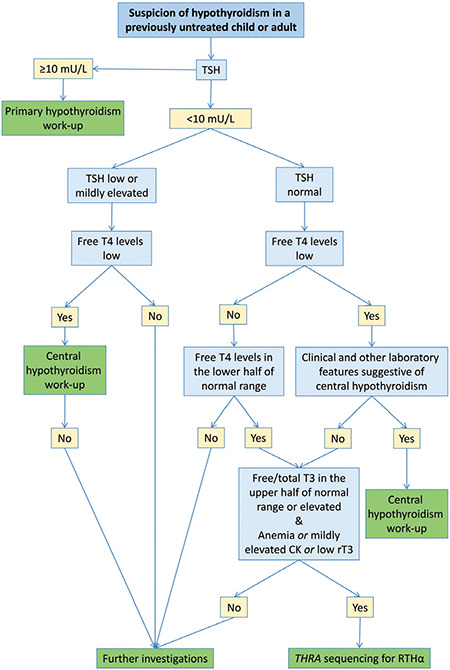
Algorithm for the differential diagnosis of hypothyroidism in previously untreated children and adults with particular emphasis on resistance to thyroid hormone alpha TSH: thyroid-stimulating hormone, RTHα: resistance to thyroid hormone alpha, CK: creatine kinase, rT3: reverse T3

RTHα should come to mind when various clinical features indicate hypothyroidism but TSH is normal and free T4 is low or in lower half of normal range in patients who have not received LT4 treatment (Figure 4). Parental medical history should be investigated thoroughly for similar clues due to autosomal dominant inheritance. More common conditions including non-thyroidal illness, recovery from thyrotoxicosis, or technical assay problems, may result in similar biochemical features (75). However, they are not associated with clinical features of RTHα.

Central hypothyroidism should be ruled out when free T4 is low and TSH is low, normal, or slightly elevated. The presence of hypothalamic-pituitary disease, hypo- or hyper-secretion of other pituitary hormones or genetic findings would indicate an etiology of central hypothyroidism (75). On the other hand, if T3 levels are elevated or close to the upper limit, the probability of central hypothyroidism is low.

Laboratory findings including elevated/normal T3, reduced rT3, normal or low T4, and normal/elevated TSH are also found in MCT8 deficiency (Allan Herndon Dudley syndrome). However, clinical and laboratory signs of peripheral thyrotoxicosis are present in this disease in addition to cerebral hypothyroidism (76,77,78,79). Furthermore, MCT8 deficiency is inherited in an X-linked manner (80). Thus, the mothers of affected patients, all of whom would be expected to be male, are asymptomatic carriers. However, an affected parent can be found in case of RTHα (16,21,23,24,25,77,78).

Additional clues for RTHα in LT4-naive children and adults are free or total T3 in the upper half of the normal range or above the upper limit, along with at least one of normocytic/macrocytic anemia *or* mildly elevated CK *or* low rT3. Among the subjects with available data, the algorithm in Figure 4 is valid for 15 of 16 children (94%) and for six of eight adults (75%) (15,16,17,21,22,23,24,25,27,28,29,30,31,32). When the data of four additional adult cases, whose assessments were available after discontinuation of LT4, are also included, the algorithm should be modified regarding T3 and TSH data, given that fT3 levels may also be in the lower half of the normal range and TSH levels can be mildly elevated. In these subjects, after exclusion of central hypothyroidism, presence of either normocytic or macrocytic anemia *or *mildly elevated CK values *or* low rT3 levels would be an additional clue leading to *THRA* sequencing. This approach is valid for 10 of 12 adult patients with available data (83%) (16,17,18,19,22,23,24,30). Both approaches require confirmation of these specificities in future studies.

## Treatment and Outcomes

There is only limited data about the treatment of RTHα and thus long-term follow-up data is required. LT4 treatment has been the first choice to date, in order to overcome the resistance in TRα with higher dosage. T4 and rT3 levels come into the normal range with this treatment and T3 level remains high. Since the feedback mechanism of the HPT axis is intact, LT4 treatment causes TSH suppression in RTHα patients (15,17,18,19,23,24,29).

In animal models with mutant TRα, increasing serum TH levels alleviated locomotor and behavioral irregularities (59). Therefore, LT4 supplementation to raise circulating TH levels was suggested to be beneficial in RTHα. Bassett et al (43) reported that prolonged T4 treatment advanced bone rigidity and strength in TRα mutant mice. However, it did not exert any effect on skeletal development, linear growth or mineralization of bones (43). Vennström et al (58) suggested that high doses of T3, given in the appropriate developmental time period, should improve the abnormalities depending on the specific mutation present in TRα. They also showed that metabolic symptoms of mice with mutant TRα, were well treated by T3. Regarding this, Espiard et al (22) reported that their case with an atypical phenotype received liothyronine treatment and a notable cardiac and metabolic response was observed. Nevertheless, other parameters did not change significantly, suggesting that the variant in this case only exhibited limited resistance to T3.

Van Mullem et al (17) reported the results of two RTHα patients (a daughter and her father, with the same variant), who were treated with LT4 for over five years. They showed that some clinical features, such as constipation or nerve conductance, were improved. However, fine motor abilities or cognitive functions did not benefit from treatment (17). On the other hand, most of the LT4 treated patients had better motor coordination, alertness, school performance, concentration or motivation (19,25,29,31). However, limited benefit on linear growth has been reported (15,17,23). Hypotonia was ameliorated and accelerated neuromotor development was observed in children (23,31,32). Thus, if the treatment was started at an early age, the benefits for development and growth would be more distinguishable. As described in the report by van Mullem et al. (17), constipation improved with LT4 treatment in most of the other RTHα cases (15,19,25,29).

With the peripheral effects of LT4 treatment, increases in sex hormone binding globulin (SHBG) or IGF-1 levels can be seen, as previously reported in RTHα patients. In addition to this, CK or cholesterol levels were reduced in these cases, reflecting the improved tissue response to TH (15,17,18,19,23,24,25). It has also been shown that when LT4 treatment was interrupted, all these indicators turned back to pretreatment levels (17). Korkmaz et al (29) reported a decrease in SHBG levels and found IGF-1 levels unaltered after LT4 treatment in a patient with RTHα, although the TSH level was suppressed and CK levels were decreased. Moran et al (18) reported a progressive rise in bone turnover markers after LT4 treatment in a case with RTHα. Growth hormone was added to LT4 therapy, due to low-normal IGF-1 levels in an affected child, but sufficient improvement in linear growth was not observed (17).

Anemia seems to be unresponsive to LT4 treatment, as described in most of the RTHα cases (18,19,25,29). Although van Gucht et al (71) showed that human erythroid progenitors responded to T3 exposure in an experimental study, they hypothesized that mutant TRα may play a role in the earlier stages of erythropoiesis, which they could not examine in their research. In addition, LT4 treatment had a limited effect on cardiac function in several cases with RTHα (18,19). Increase in heart rate was observed in one patient after LT4 treatment (22).

Patients who had frameshift variants in *THRA*, including the carboxy-terminal part of TRα1, had varying responses to LT4 treatment. Like their severity of clinical presentation, this situation was also associated with the specific location of the variant or the degree to which this molecular region was affected (17,18,24). In patients with frameshift variants skeletal abnormalities did not respond to LT4 treatment (17,18,24).

Since LT4 administration to RTHα patients will excessively stimulate TRβ in TRβ-dominant tissues, development of TRα1-selective thyromimetics would be ideal (33,81). Alternative investigations targeted HDAC activity or interaction with the co-repressor complex to inhibit the dominant-negative effect of wild-type analogue of mutant TRα1. It was shown in a murine study that a mutation in NCoR can disrupt its co-action with TRα1 and reverses the effects of mutant TRα (82). An HDAC inhibitor, suberoylanilide hydroxamic acid (SAHA), was used to relieve the repression in target genes and phenotypic features improved in TRα1 mutant mice (81,83,84). However, Freudenthal et al (38) showed that SAHA was unlikely to treat skeletal abnormalities and had no effect on bone structure or strength in TRα mutant mouse models. These authors suggested that alternative co-repressors, in addition to NCoR, may interact with TRα in skeletal cells (36,38).

## Conclusion

The diagnosis of RTHα is not straightforward since TH levels might not be helpful and the entity is not widely known. As published data is limited concerning RTHα, absence of phenotypic features or laboratory findings would not exclude RTHα. Currently, only fT4 and TSH levels are recommended for evaluation of growth failure in children (85). However, these tests can be normal in a subject with RTHα and astute clinicians should do further investigations in such a case when the clinical picture is similar to hypothyroidism. In addition, RTHα should be kept in mind in patients diagnosed with apparent central hypothyroidism, particularly when the exact etiology cannot be determined.

## Figures and Tables

**Table 1:**
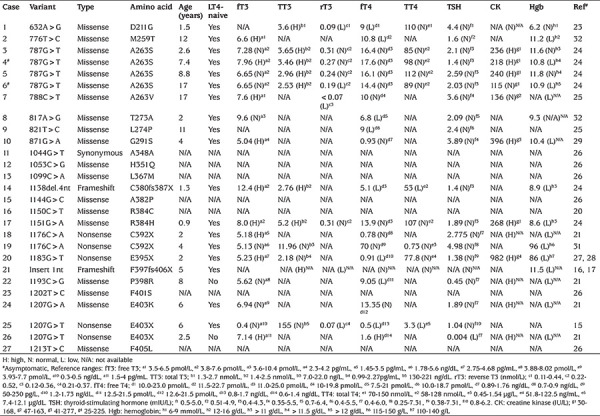
Genetic and laboratory findings in reported children with *THRA* variants (n=27). Except for two subjects, all of the patients with available data had at least one symptom or sign associated with hypothyroidism. Laboratory data were obtained before LT4 use in all subjects except patients 22 and 26, who were receiving LT4 treatment. When available, the data were given as exact values [high (H), normal (N), or low (L)] and relevant reference ranges in the original reports were given as footnotes

**Table 2:**
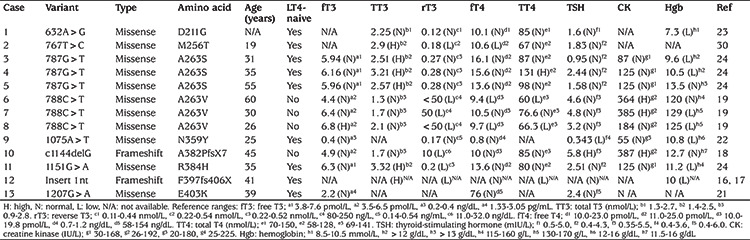
Genetic and laboratory findings in reported adults with resistance to thyroid hormone alpha ( n=13). All of the patients had at least one symptom or sign associated with hypothyroidism. The data of patients 6, 7, 8, and 10 were obtained after discontinuation of L-thyroxine (LT4), which was used for many years. Remaining laboratory data were obtained before LT4 use. When available, the data were given as exact values [high (H), normal (N), or low (L)] and relevant reference ranges in the original reports were given as footnotes

**Table 3:**
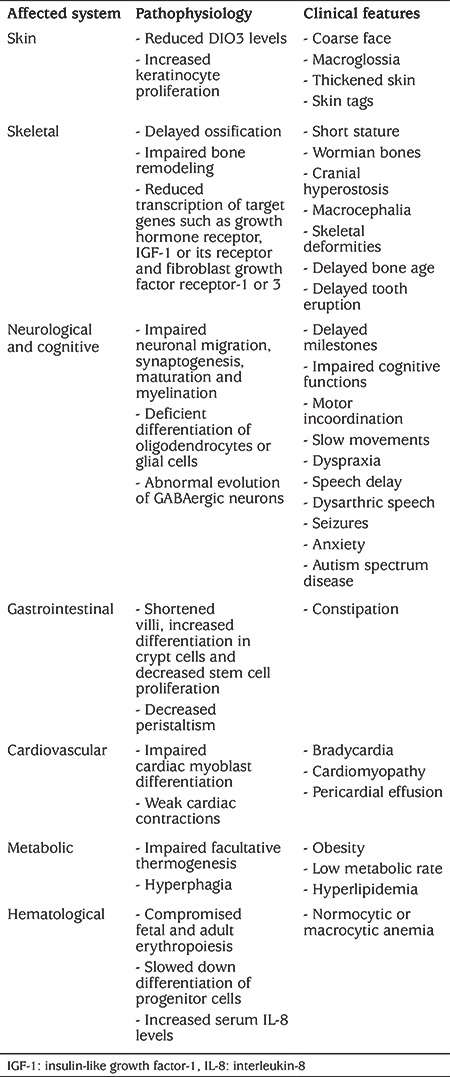
Summary of clinical features and underlying mechanism for resistance to thyroid hormone alpha. Pathophysiological mechanisms were observed from animal models, except for hematological findings
